# Targeted design of a recombinant tracer for SPECT renal imaging

**DOI:** 10.7150/thno.60132

**Published:** 2021-08-27

**Authors:** Pan Liu, Steven E. Johnson, Xinfang Xie, Li Gao, Chad R. Haney, Ming Zhao, Jing Jin

**Affiliations:** 1 Division of Nephrology and Hypertension, Feinberg School of Medicine, Northwestern University, Chicago, IL, USA.; 2 Feinberg Cardiovascular and Renal Research Institute, Feinberg School of Medicine, Northwestern University, Chicago, IL, USA.; 3 Center for Advanced Molecular Imaging, Northwestern University, Chicago, IL, USA.; 4 Department of Nephrology, The First Affiliated Hospital of Medical College, Xi'an Jiaotong University, Xi'an, China.; 5 Department of Cardiology, The First Affiliated Hospital of Medical College, Xi'an Jiaotong University, Xi'an, China.

**Keywords:** Nuclear Medicine Imaging, Renal scan, Single-photon emission computed tomography (SPECT), Radiotracer, Recombinant protein probe, Neonatal Fc receptor (FcRn)

## Abstract

**Rationale:** A robust radiopharmaceutical has high uptake in the target and low retention in non-target tissues. However, traditional tracers for renal imaging that chemically chelate ^99m^Tc are excreted through the renal route with transient resident time in the kidney. Following a rational design approach, we constructed a protein-based radiotracer, designated PBT-Fc, to sequentially bind tubular neonatal Fc-receptor and subsequently proximal tubular basement membrane for its targeted sequestration in kidney parenchyma. In this process, the tracer participates in physiologic glomerular filtration and tubular reabsorption while escaping lysosomal catabolism and urinary clearance.

**Methods:** To specifically target renal receptors in navigating the urinary passage in the kidney, we produced a recombinant fusion protein with two separate functional parts: a polybasic PBT segment derived from human Vascular Endothelial Growth Factor and Fc segment of IgG1. The chimeric fusion of PBT-Fc was labeled with radionuclide^ 99m^Tc and tested in rodent models of kidney diseases. Planar scintigraphy and single-photon emission computerized tomography (SPECT) were performed to evaluate renal-specificity of the tracer.

**Results:** When injected in mouse and rat, following a brief 10 - 15 min dynamic redistribution phase in circulation, ~ 95% of the [^99m^Tc]-PBT-Fc signal was concentrated in the kidney and lasted for hours without urinary loss or surrounding tissue activities. Long-lasting tracer signals in the kidney cortex in conjunction with SPECT greatly augmented the image quality in detecting pathological lesions in a variety of disease models, including ischemic acute kidney injury, drug-induced renal toxicity, and chronic kidney disease from renin-angiotensin system (RAS) overactivation.

**Conclusion:** Exclusive renal retention of the recombinant radiotracer greatly facilitated static-phase signal acquisition by SPECT and achieved submillimeter spatial resolution of kidney alternations in glomerular and tubular disease models.

## Introduction

Avid radiopharmaceuticals such as ^123^I and ^131^I in thyroid scan, methylene diphosphonate (MDP) in bone scan and neurotransmitter analogs in brain imaging are involved in human metabolic processes with high levels of sequestration in the target organs 1, 2. Current tracers used in renal scan diagnostic imaging, such as diethylenetriaminepentaacetic acid (DTPA), mercapto-acetyl-triglycine (MAG3), dimercaptosuccinic acid (DMSA), and glucoheptonate (GHA) are radionuclide-chelates 3-9 that, depending on the main route of their renal-urinary clearance, each provides a slightly different advantage over the others to assess renal function or anatomy 10, 11. For instance, DTPA is primarily cleared by renal filtration and used in renal scintigraphy for measuring filtration dynamics 12, 13, while MAG3 enters the urinary tract mostly via tubular excretion and is used to detect urinary tract obstruction (in Lasix scan) 14-16. In contrast, DMSA and GHA have longer tissue retention in the kidney and in other organs, and are therefore selected for obtaining static images in SPECT 9, 10, 17. Nevertheless, all conventional radiotracers have transient and dynamic resident time in the kidney that are not ideally suited to achieving high spatial resolution 15. Due to this limitation, existing nuclear medicine modalities cannot provide detailed anatomic information about renal lesions, particularly those that affect the glomerulus and/or the renal tubule 3, 10, 16, 18.

One of the unique characteristics of the kidney is the highly organized spatial pattern of its cortical filtering system, transporters, and receptors, along ~ 1 million individual nephrons that convert blood filtrate to urine. In addition to water and electrolytes, plasma proteins in the filtrate are distinctively sorted and transported by their cognate tubular receptors 19, 20. We purposely selected the IgG fragment crystallizable (Fc) domain and VEGFA extracellular matrix (ECM) binding domain in constructing chimeric fusion protein to use as imaging tracer for examining renal functions. In accordance with the design, after *i*.*v*. injection in rodents, the tracer protein followed a distinct path in the kidney based on its molecular size and receptor-binding specificity. The *in vivo* characteristics of the tracer indicated its suitability for nuclear imaging purposes. These attributes include the participation of the tracer in glomerular filtration, tubular reabsorption, and more importantly, a long-lasting sequestration of the tracer in the kidney without significant signal loss due to lysosomal degradation or urinary excretion.

## Materials and Methods

### Construction of the tracers

The polybasic tag (PBT) sequence was derived from human VEGFA, protein sequence accession: NP_001020537.2, amino acids 131 - 163. The Fc fragment was derived from human IgG1/IGHG1 heavy chain sequence from amino acids 239 - 470, and was reported previously 21. The PBT and Fc modules were joined by in-frame fusion of their encoding cDNA fragments, which were cloned into pET-30a vector (Millipore-Sigma) with an N-terminus 6xHis tag as an extension. The Fc alone fragment was also cloned into pET-30a vector as control.

### Recombinant production

Each plasmid was transformed into BL21 DE3 strain of *E. coli* for recombinant expression of the tracer protein. In general, induction was performed with 400 μM of IPTG at 22 °C for 16 h. Lysis was assisted with sonication of the bacterial homogenates on ice for 30 min in binding buffer containing 20 mM sodium phosphate (pH 7.4), 0.5 M NaCl and 20 mM imidazole. Following centrifugation, the soluble fraction of the lysate was collected and loaded onto a HisTrap HP column via an FPLC system (GE Life Sciences). Purified tracer proteins were subjected to endotoxin removal using endotoxin removal spin columns (Pierce/ThermoFisher), and then desalted using Zeba™ desalting columns (7K MWCO, Pierce/ThermoFisher). The tracer proteins were then maintained in phosphate-buffered saline (PBS, pH 7.4) at 1 mg/mL and stored in -80 °C as 0.5 mL aliquots.

### HYNIC conjugation and ^99m^Tc labeling

S-HYNIC (Vector Laboratories) was freshly dissolved in dimethylformamide to a final concentration of 10 mg/mL. 3 μL of the HYNIC solution was added to 0.5 mL of the tracer protein solution to start the reaction, which lasted for 2 h at room temperature under gentle agitation. The mixture was then buffer exchanged into 20 mM citrate and 100 mM NaCl buffer at pH 5.2 using Zeba™ desalting columns. Then 75 μL of tricine solution (100 mg/mL dissolved in the citrate buffer) was added to the HYNIC-labeled tracer protein. In the morning of SPECT, ^99m^Tc pertechnetate was added to the above solution immediately followed by adding 20 μL of stannous chloride solution (10 mg/mL in 0.1 N HCl). The labeling reaction was allowed to proceed for 1 h at room temperature. Following the reaction, the tracer was buffer exchanged into PBS using Zeba™ desalting columns, and the flow-through of ^99m^Tc-HYNIC-labeled tracer was measured for radioactivity.

### Neonatal Fc receptor binding assay

Binding affinities of PBT-Fc and PBT-Fc-HYNIC to Neonatal Fc receptor (FcRn) were measured by Lumit™ FcRn Binding Immunoassay kit (Promega) following the manufacture's instruction. Purified human IgG and IgA (purchased from Millipore) were used as positive and negative controls, respectively.

### Pharmacokinetic analyses

The pharmacokinetics of Fc and PBT-Fc were assessed in female Balb/c mice. Both proteins were labeled by Fluorescein isothiocyanate (FITC, Sigma) following the manufacture's instruction. The mice received *i.v.* injection of Fc-FITC (25 μg/mouse) or PBT-Fc-FITC (56 μg/mouse) (equalized at the same total fluorescence for Fc-FITC and for PBT-Fc-FITC). Blood samples were collected from tail bleeding at a number of indicated timepoints following injection. Collected blood samples were immediately diluted 10 times in PBS buffer with 5 mM EDTA and then measured by fluorescence spectrophotometers at Ex_485_/Em_530_ nm wavelengths for measuring tracer concentrations.

### Radio scintigraphy of rats

Anesthetized rats (induced by 4% isoflurane and maintained with 2% isoflurane) were scanned using a clinical planar γ-camera (Model XRT, GE Healthcare). For each rat, approximately 37 MBq of total radioactivity of the probe was injected via the tail vein. Dynamic radio signals were collected continuously for the duration of 60 min. Frame-per-minute images captured the dynamic redistribution of the *in vivo* tracer and the static image was taken by combine the steady phase signals between 40 and 60 min.

### Micro-SPECT/CT imaging of mouse

SPECT was performed with a U-SPECT+/CT (MILabs) on anesthetized mice. The anesthesia was induced by 4% isoflurane and maintained with 2% isoflurane throughout the imaging process. For each mouse, 11 MBq of the tracer was injected, and data were collected using a collimater with 0.6 mm pinholes and 0.4 mm resolution, and sensitivity was set at > 1,500 cps/MBq. A 15 min acquisition (for ^99m^Tc-PBT-Fc) or 10 min acquisition (for ^99m^Tc-MAG3 and ^99m^Tc-DMSA) was reconstructed. Reconstructed voxel sizes were 0.2 mm. MicroCT was used for attenuation correction and anatomic guidance.

### Renal disease models of mice

Unilateral ischemia reperfusion (IRI) was performed on 8 weeks old male Balb/c mice following a standard surgical procedure. In brief, mice were anesthetized by *i.p.* injection of Xylazine/Ketamine mixture and warmed on heating pad during the following procedure. An abdominal incision was made to expose the left kidney, the renal pedicle was bluntly dissected and clumped by a non-traumatic microvascular clip for the indicated length of time. After the clip was removed to restore blood circulation to the kidney, we closed the wound with suture and administrated analgesic (SR-buprenorphine, 1 mg/kg, SC). We also administered 0.5 mL warm saline subcutaneously to prevent dehydration of the animals. Mice were then placed on the heating pad until they recovered.

The Adriamycin nephropathy model of glomerular injury were generated with 8 weeks old male Balb/c mice. A single dose of Adriamycin (11 mg/kg BW) was *i.v.* injected in mice via the tail vein. Following injection, body weight was monitored daily. Mice that had lost > 20% of the initial body weight were selected for SPECT imaging study 5 days following the treatment. The folic acid nephropathy model was created by *i.p.* injection of folic acid (250 mg/kg BW) to 8 weeks old Balb/c mice, and the animals were imaged 3 days after. The chronic hypertensive model of transgenic mice, RenTgMK, developed chronic kidney dysfunction at old age. 8 months old homozygous renin-transgenic mice were selected for SPECT and renal histology studies.

### Immunohistochemistry and immunofluorescence microscopy

Preparation of cryosections for immunohistochemistry (IHC) and immunofluorescence (IF) was performed with the kidneys. The tissues were fixed in 4% PFA for overnight at 4 °C followed by incubation in 20% sucrose solution for the purpose of cryoprotection. The tissues were then embedded in OCT and microscopic sections were prepared at 7 μm thickness using a cryostat machine. For detecting the PBT-Fc tracer in the kidney by IHC, the sections were stained with HRP-conjugated goat anti-human IgG antibody and developed in DAB substrate. For IF detection of the tracers, the sections were stained with Dylight-595 labeled goat-anti-human IgG Fc antibody (ThermoFisher). To label the tubular basement membrane, the sections were co-stained with Rabbit anti-Col4a1 antibody (ThermoFisher) followed by Alexa-488-labeled goat-anti-rabbit secondary antibody (ThermoFisher).

### Fitting PBT-Fc's renal sequestration dynamics with theoretical glomerular filtration rate

Following intravenous injection of rats using [^99m^Tc]-PBT-Fc, continuous whole-body scanning by planar (coronal) gamma camera captured the redistribution dynamics of the radiotracer, namely a graduate accumulation of kidney signals. The accumulation of signal pixels in the kidney areas as a percentage of the whole-body levels (plotted in *y*-axis) was calculated for the selected timepoints (*x*-axis). These timepoint values were compared to a theoretical curve of renal sequestration of the probe away from circulation over the time. The theoretical curve was based on an assumed glomerular filtration rate (GFR) of 5 mL/min (9.6 mL/min/kg) as measured by Katayama et al 22 with a total blood volume of 25.6 mL in rats 23. Unlike traditional GFR tracers, such as iodixanol, which is filtered at the glomerulus and then excreted into the urine, PBT-Fc tracer does not follow urinary excretion after been filtered. This is despite that supposedly both iodixanol and PBT-Fc are cleared from blood circulation via glomerular filtration at comparable rates. Therefore, iodixanol clearance through the kidney 22 and PBT-Fc accumulation in the kidney as function of time are expected to follow a standard differential equation of *y* = [v-v*e(-f/v*x)], in which *y* is the total amount in percentage of tracer clearance from blood, or total tracer accumulation in kidney in the case of PBT-Fc; *v* is the total blood volume of a rat; *e* refers to as log(*e*) = 0.4342; *f* is kidney filtration rate/GFR in mL/min; and *x* is the total time span since injection in min. Based on this differential equation for calculating blood clearance rate, the theoretical rate of PBT-Fc sequestration in the kidney is plotted (total renal signal in the *y*-axis against total time span in the *x*-axis following the injection of the tracer), provided that the tracer did not leave the kidney during the 30 min of recording.

### Statistical analysis

The data are reported as mean ± SE. Differences between 2 groups were analyzed using a two-tailed Student's t-tests. GraphPad Prism software was used for all statistical analyses, and a *p* value of less than 0.05 was considered significant.

## Results

### The design of PBT-Fc tracer for renal sequestration

**Figure 1:**
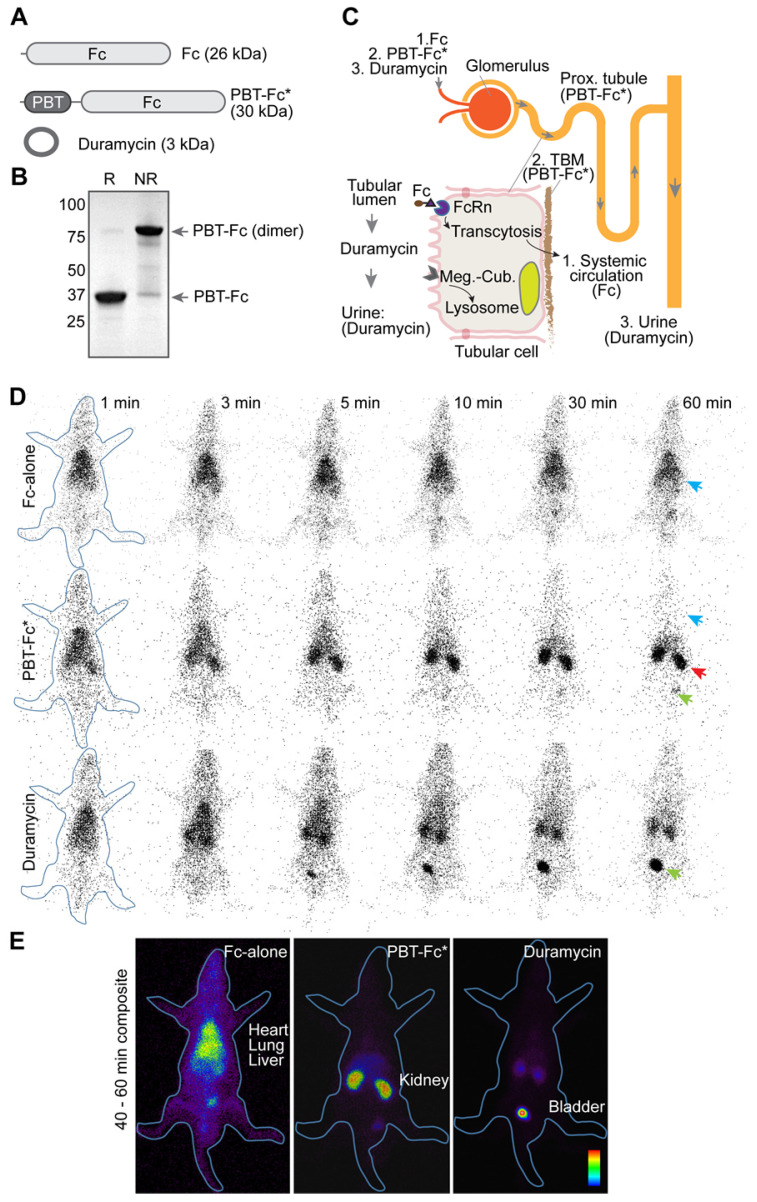
***in vivo* testing of recombinant radiotracers constructed by joining protein modules of PBT of VEGFA and Fc of IgG1**. **A.** The domain architectures of constructed protein tracers, of which chimeric fusion PBT-Fc was selected for in-depth studies (highlighted by an asterisk). **B**. PBT-Fc expressed from *E. coli*. naturally existed as a dimer, as shown on SDS-PAGE under non-reducing condition (NR) when compared to reducing condition (R). **C**. Schematic illustrations of nephron (upper right) and proximal tubular epithelial cell (bottom left inset). The distinct intrarenal passages of individual tracers (1: Fc-alone; 2. PBT-Fc; and 3. Duramycin) including their interactions with corresponding cell receptors and ECM target(s) are shown (follow arrows). The steady phase location of each tracer is indicated by parentheses. **D**. Selected minute-by-minute planar scanning images of [^99m^Tc]-labeled Fc-alone, PBT-Fc and duramycin in anesthetized rats, showing distinct dynamic patterns of these tracers. Apparent heart/lung blood pool (blue arrow), the kidney (red arrow) and the bladder (green arrow) locations are indicated. **E**. Combined steady phase images between 40 and 60 min showed contrasting differences of systemic circulation, renal sequestration, and urinary excretion profiles for Fc-alone, PBT-Fc and duramycin tracers, respectively. The experiment was repeated 3 times and representative images are shown (n = 3).

The principle component of our probe is Fc segment of IgG1 (Figure 1A), which binds neonatal Fc receptor (FcRn). In the kidney, FcRn is responsible to reuptake IgG and albumin that are inadvertently filtered at the glomerulus 24. FcRn then mediates transcytotic recycling of its “cargo” proteins back to systemic circulation as intact proteins, free from lysosomal degradation 25, 26. We considered using Fc-based tracers to probe for these glomerular and tubular activities. To this end, we made recombinant proteins using either Fc-alone 26, or as a fusion with a 33 amino acid segment of Vascular Endothelial Growth Factor A/VEGFA-165 (designated polybasic tag, or PBT; details in Methods) (Figure 1B) that displays an affinity to tubular basement membrane (TBM) matrix (unpublished observation). As expected, in our *in vitro* tests, this PBT-Fc tracer showed a specific affinity to FcRn (as compared with precursor protein IgG as a positive control; and IgA as a negative control), with an IC_50_ of 0.23 nM (Figure S1). Similarly, the HYNIC modified PBT-Fc also exhibited comparable affinity to FcRn (0.33 nM), confirming the targeting specificity of our recombinant tracer. Accordingly, Fc-alone probe constantly cycles through systemic circulation, followed by glomerulus filtration, and then by tubular reuptake and recycling back to circulation via receptor (FcRn)-mediated recycling by the kidney proximal tubule (Figure 1C). In contrast, while PBT-Fc fusion is still freely filtered at the glomerulus and subsequently transported by FcRn across the tubular epithelial layer, the tight interaction between PBT and the TBM matrix prevents the tracer molecule from reentering circulation via the peritubular capillary (Figure 1C). Over time, there is a gradual accumulation of the PBT-Fc signal within the kidney while the tracer is being cleared from systemic circulation.

### PBT-Fc tracer is highly enriched in the kidney following *i.v.* injection

We sought to test the design concept in live imaging of rats with radionuclide ^99m^Tc-labeled tracers of Fc-alone, PBT-Fc, and a short peptide control duramycin. Each rat was injected with 37 MBq of tracer via the tail vein. Dynamic whole-body planar images were acquired by a gamma camera at 1 min/frame for a total duration of 60 min. As expected, the whole-body imaging data unequivocally showed that [^99m^Tc]-PBT-Fc exhibited a rapid, time-dependent accumulation in the kidney between 1 to 10 min following *i.v.* injection (Figure 1D). Thereafter, the kidney signals remained mostly unchanged between 10 min to 60 min, indicating near-complete renal retention of PBT-Fc with minimal washout. By contrast, the Fc control remained predominantly in blood circulation without selective renal accumulation (Figure 1D). Steady 'blood pool' signals could be visualized in richly perfused organs, such as the lung, the heart and the liver. Pharmacokinetic studies also showed contrasting difference between Fc-alone and PBT-Fc, with Fc-alone stayed in blood and PBT-Fc quickly disappeared from circulation (Figure S2), consistent with the results of gamma scanning. In addition, duramycin underwent fast renal-urinary excretion to the bladder without prolonged renal retention (Figure 1D), expected to resemble the standard of care tracer DTPA in renal scan. As compared to Fc-alone and duramycin controls, it was apparent that PBT-Fc was enriched in the kidney with minimal levels of non-renal signals and no probe loss due to further urinary excretion. The exclusive renal sequestration of PBT-Fc during the static phase of bio-distribution greatly facilitated signal acquisition of the kidney for spatial mapping of nephron activities (signal collection for 20 min in Figure 1E compared to 1 min exposure in Figure 1D).

**Figure 2:**
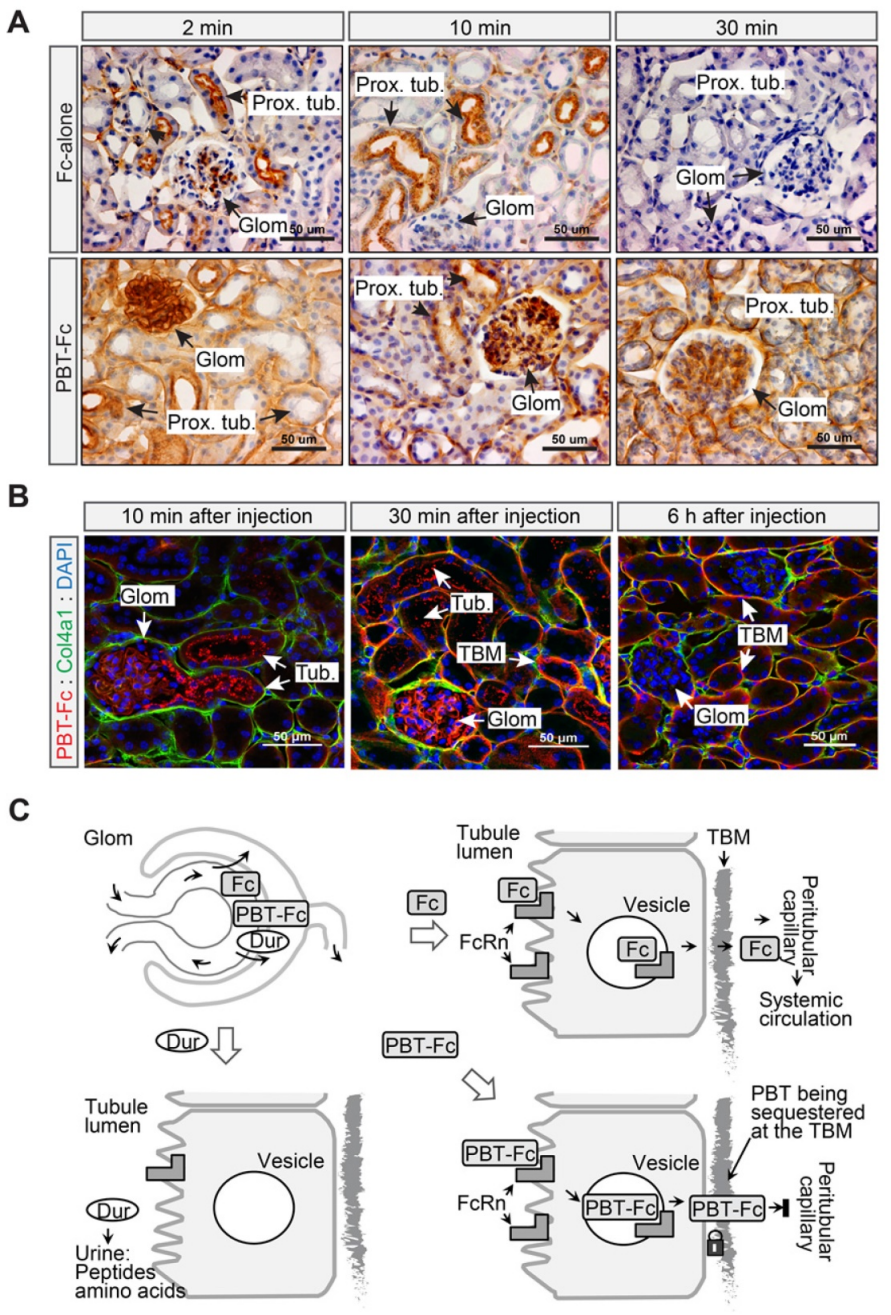
** The PBT tag contributed to tubular basement membrane retention of PBT-Fc. A**. 2 to 30 min following *i.v.* injection, Fc-alone passed through the glomerulus, the proximal tubule, and was then maintained a low level in the kidney as shown by immunohistochemistry staining against human Fc. Meanwhile, PBT-Fc followed a time-dependent accumulation, first in the glomerulus (2 min), followed by in tubular cell body (2 min and 10 min), and lastly in tubular basement membrane (TBM: 30 min). **B**. As shown by immunofluorescence, PBT-Fc was firstly filtered at the glomerulus (Glom. 10 min) to enter the proximal tubule (Tub.). By 30 min, the tracer was localized predominately to TBM and GBM. By 6 h majority of PBT-Fc was associated with TBM. Scale bar: 50 µm. **C**. A schematic representation of the distinct cellular passages of duramycin (Dur: bottom left), Fc-alone (Fc: top right) and PBT-Fc (bottom right). The schematics focus on the role of canonical Fc receptor FcRn on tubular epithelial cells to intact “cargo” proteins across the epithelial layer. Upon reaching TBM at the basal side of epithelium, Fc-alone reenters systemic circulation via peritubular capillary, whereas PBT-Fc is sequestered at the TBM.

Next, we performed a time series IHC and IF study to verify the intrarenal route of PBT-Fc (Figure 2). Mice were *i.v.* injected with either Fc-alone or PBT-Fc, and kidneys were harvested at 2 min, 10 min, 30 min, and 60 min time points. Sections of the kidneys were stained with anti-human Fc antibody. IHC showed contrasting dynamics of the two tracers in the kidney cortex (Figure S3). Fc-alone had a scattered presence at 2 min and 10 min. The renal staining then disappeared, consistent with the speculation that Fc-alone control probe stayed in blood circulation without the sequestration in the kidney. At higher magnification (Figure 2A, top left), Fc-alone at 2 min appeared in the glomerulus and the luminal side the proximal tubule, indicating filtration and tubular binding. As expected, the glomerular staining subsided after 10 min and staining of the proximal tubule cell body became more evident (Figure 2A, top middle), and by 30 min neither glomerular nor tubular staining was visible (Figure 2A, top right). In contrast, the tracer signal of kidney-targeted PBT-Fc was much stronger than that of Fc-alone, and the overall signal intensity gradually enhanced with major staining of the glomerulus shifting to tubular structures (Figure S3). While both glomerulus and tubular staining was intense, by 30 min there was a prominent staining of the tubular basement membrane (TBM) (Figure 2A, bottom right), as expected. This was further confirmed by immunofluorescence co-staining with collagen 4α1 that marks the TBM (Figure 2B). Following a single injection, PBT-Fc remained detectable at the TBM after 6 h (Figure 2B, right). Collectively, these results indicate distinct sorting of Fc, PBT-Fc and duramycin by the tubular epithelium into blood circulation, tubular basement membrane of the kidney and urine in bladder, respectively, after each being filtered at the glomerulus (Figure 2C). Also, in agreement with the radio-scintigraphy results showing predominant sequestration of PBT-Fc in the kidney (Figure 1E), a survey of steady-phase biodistribution by IF staining of multiple organs indicated the strongest signal in the kidney, much weaker signal in the spleen and the liver, and no staining of PBT-Fc in the heart and the lung, as expected (Figure S4).

### High resolution renogram imaging using [^99m^Tc]-labeled PBT-Fc tracer in conjunction with SPECT

**Figure 3:**
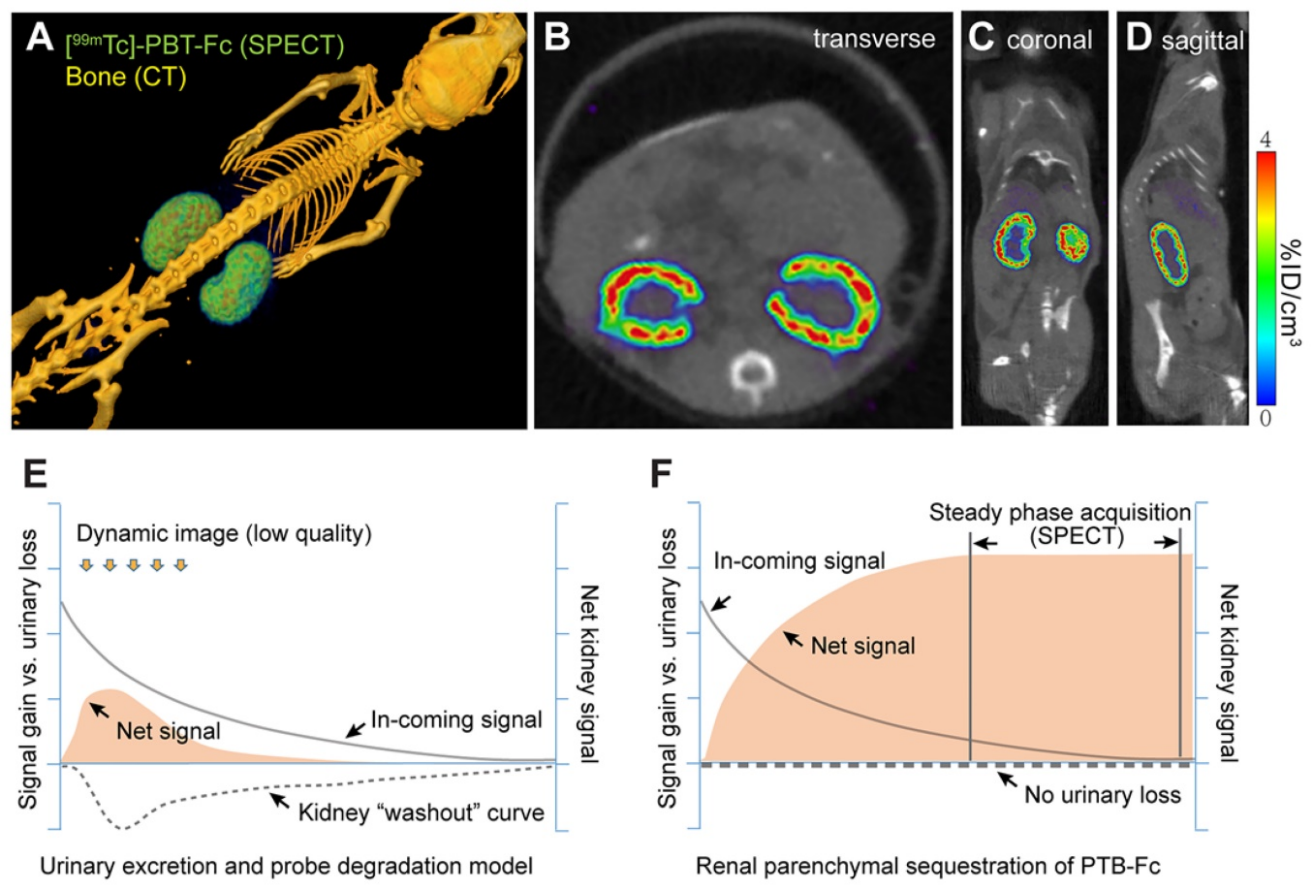
** The renal-targeting [^99m^Tc]-PBT-Fc tracer facilitated steady phase signal acquisition for SPECT**. **A**. Micro-SPECT/CT imaging of mouse, showing exclusive renal signals of [^99m^Tc]-PBT-Fc in 3-dimentionalreconstruction. Heatmap scale in %ID/cm^3^. **B-D**. Sectional views of the radiotracer enriched in kidney cortex. **E**. A schematic model for concurrent tracer signal accumulation (solid curve), and loss, in the kidney of a conventional tracer that passes through the urinary route, either due to direct excretion or caused by tracer degradation (signal “washout”: dotted line). Consequently, the net renal signal (solid fill) stays at a low level. **F**. An illustrative model for PBT-Fc that enters (solid curve) the kidney cortex with no urinary loss (dotted line) results in a gradual accumulation of renal signals (solid fill). Once reached the steady phase of signal sequestration in the renal parenchyma, there is an extended window of time for SPECT image acquisition from collecting stable radionuclide signals for developing high quality images of the kidney.

Having achieved the important benchmark of total renal sequestration with [^99m^Tc]-PBT-Fc, we then performed live micro-SPECT/CT on mice and collected static phase signals between 45 - 60 min after injection of the radiotracer. With > 95% of total signals accumulated in the kidney, high-resolution images of the kidney cortex were developed (Figure 3A). The SPECT image reflected the accumulated parenchymal activities of glomerular filtration in conjunction with tubular reabsorption that led to subsequent TBM retention of the tracer. It is noted that although continuous signals were presented across the entire renal cortex, there was an uneven distribution of signal intensity (Figure 3B-D), possibly attributable to physiologic regulation of individual nephron activity at a given time. This was reflected in both 3D-reconstructed (Figure 3A) and sectional (Figure 3B-D) views. These high-quality images at unprecedented submillimeter resolution indicated that PBT-Fc was well-suited modality for SPECT imaging of the kidney that would benefit from high levels of static phase signals. Conventional tracers, such as MAG3 and DMSA, were both quickly filtered through the kidney and dynamically excreted into the urine, and therefore could not provide sustained kidney signals (Figure 3E and Figure S5). Meanwhile, PBT-Fc had no urinary loss and thus could reach high steady-state signals in the kidney (schematic comparison between Figure 3E and Figure 3F) that are critical for SPECT imaging.

### [^99m^Tc]-PBT-Fc-directed renogram imaging of ischemia-reperfusion injury

**Figure 4:**
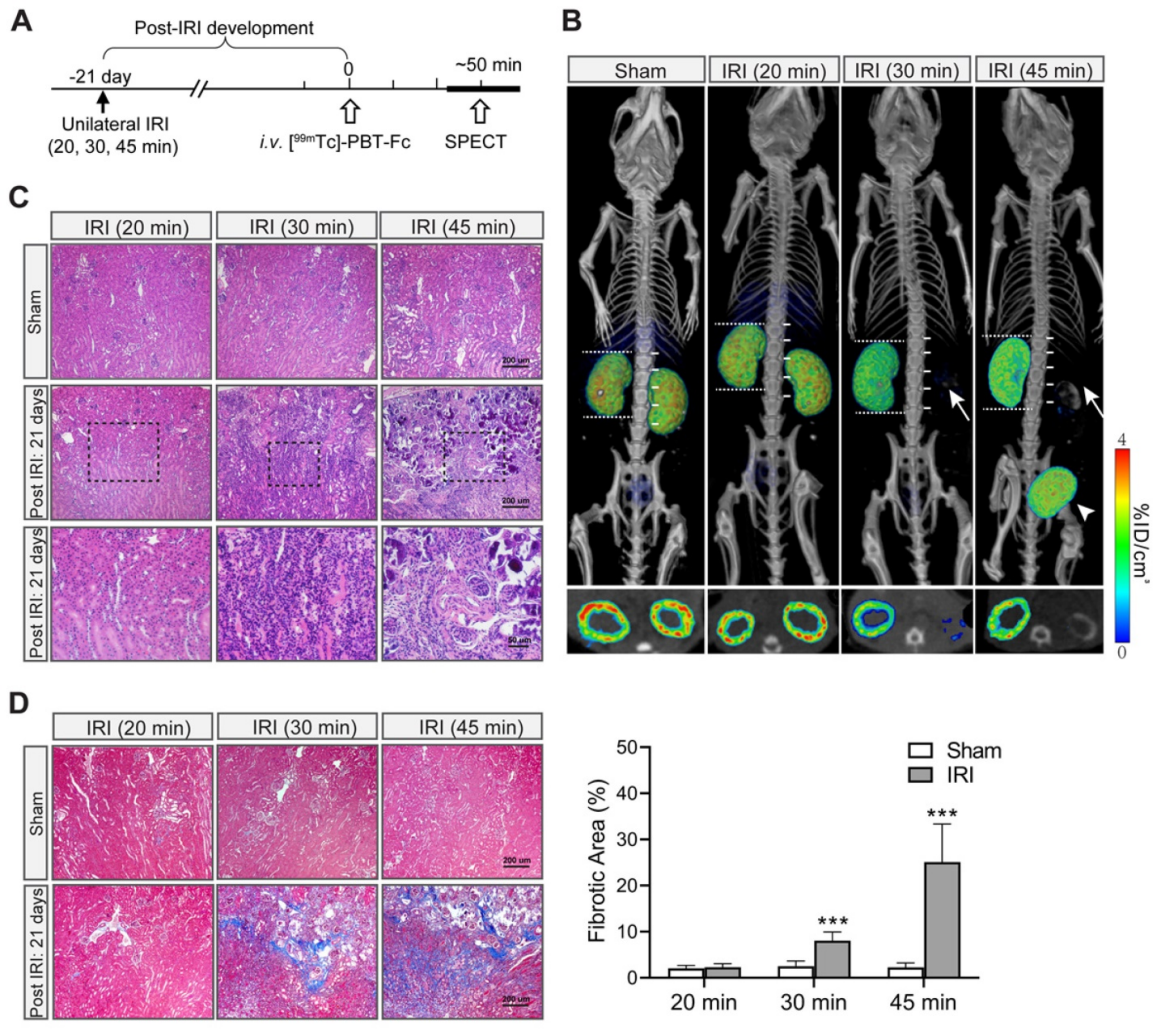
** [^99m^Tc]-PBT-Fc-directed renal SPECT revealed functional impairments in an ischemia-reperfusion injury model of mice**. **A**. Unilateral ischemia-reperfusion injury (IRI) models were generated with a controlled gradation of severity following either 20 min (mild), 30 min (medium) or 45 min (severe) clamping of renal pedicles. The animals were allowed to recover from IRI for 21 days before subjected to tracer injection and, subsequently, SPECT (workflow shown in the top panel). **B**. Images of the radiotracer in the kidney and the bladder are shown below (with composite coronal view on top and corresponding transverse view further below). The kidney operated on with mild IRI of 20 min had fully recovered. In contrast, the kidneys that had incurred more severe injuries (i.e. IRI for 30 or 45 min) had lost most of their signals (arrows). Instead, bladder accumulation of the radiotracer was evident (arrowheads). Moreover, with functional loss of one of the two kidneys, the uninjured kidney appeared larger in size (with their length measured against number of spinal cord segments), indicating compensatory growth of renal mass. Bottom panels show corresponding sectional views of the presence or loss of radionuclide tracer signals in renal cortex. Heatmap scale in %ID/cm^3^. **C**. H&E histology of IRI injured kidney showed extensive tissue damage that resembles the pathology of acute kidney injury. The sham controls were from uninjured contralateral kidney of the same animal. Both 30 min IRI and 45 min IRI showed tubular damages, ranging from lymphocyte infiltration (middle and right panels), to tissue necrosis (right panels). The tubular structures of 20 min IRI model appears normal (left panels). The bottom panels are selected insets (dotted boxed) at higher magnification. **D**. Masson trichrome stain of sham vs. IRI kidneys (n = 3). The 30 min and 45 min IRI kidneys showed extensive staining signals for interstitial fibrosis. Little fibrosis was present in the 20 min IRI kidneys (compared to sham surgery kidneys). Tissue fibrosis was semi-quantified from 10 randomly chosen images in each group. ***, p < 0.001.

Next, we tested [^99m^Tc]-PBT-Fc on an ischemic-reperfusion injury (IRI) model 27 to detect functional renal loss versus recovery responses. Mice were subjected to a surgical operation of unilateral IRI of the right kidney with a range of severities and then allowed to recover for 21 days (Figure 4A). [^99m^Tc]-PBT-Fc-directed SPECT illustrated various responses of the injured kidneys. By comparing the preservation of radiotracer signals in the injured versus contralateral control kidneys (Figure 4B, right compared to left kidneys, respectively), the severity of renal impairment was determined. Notably, kidneys were full recovered from a mild IRI (such as 20 min). In contrast, 30 min IRI irreversibly damaged the kidney with the loss of most of its tracer signals (Figure 4B). 45 min IRI caused further reduction of signal retention accompanied with radionuclide leaking into the bladder (Figure 4B, right panel). This may be due to pathologic protein leakage from the injured kidney with impaired tubules that is reminiscent of acute kidney injury (AKI) in patients 28. In addition, following the unilateral kidney impairment as in the examples of 30 min and 45 min IRI, the uninjured kidneys had apparently increased their sizes (Figure 4B, as measured against spine vertebrae), indicating compensatory growth of renal mass, and presumably function capacity, of the healthy kidneys. The signal loss in the severe IRI kidneys, as seen in 30 min and 45 min ischemia, was consistent with histologic findings of the kidneys with extensive lesions of tubular damage, lymphocyte infiltration, tissue necrosis and interstitial fibrosis 21 days after the initial IRI (Figure 4C-D).

### Additional models of acute kidney injury imaged using PBT-Fc tracer

**Figure 5:**
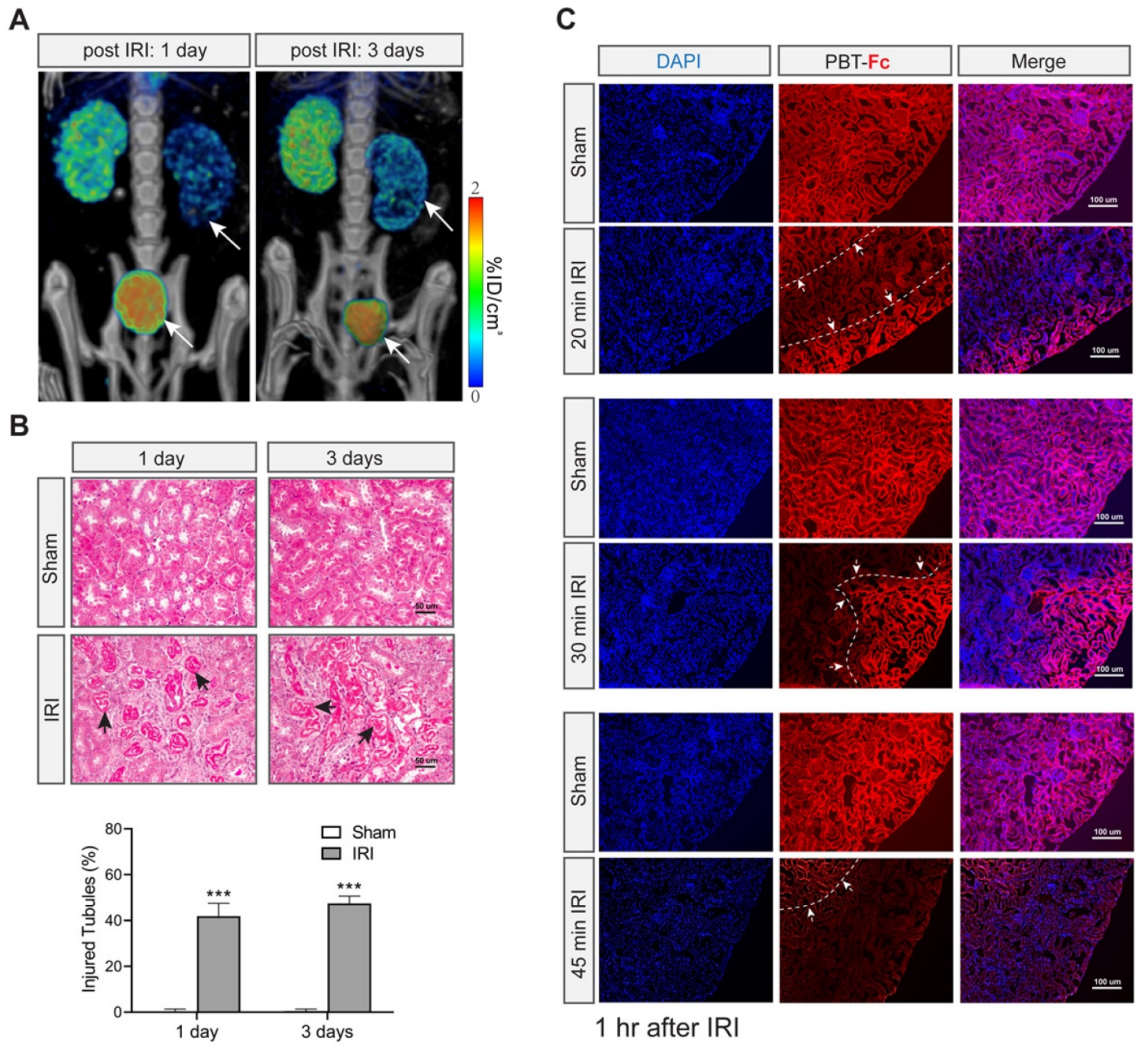
** [^99m^Tc]-PBT-Fc detects acute kidney injury in mice. A.** Post IRI injury (with 30 min clamping) as early as 1 and 3 days already showed reduced tracer signals (arrows) as compared to the contralateral healthy kidneys. Concurrent bladder signal (arrowheads) of the tracer was also observed, likely attributed to tracer leakage from the injured kidneys. Heatmap scale in %ID/cm^3^. **B**. Masson trichrome stain of sham and IRI surgery kidneys showed no fibrosis in these acute stages (1- and 3- days post-surgery). However, extensive tubular damage was observed as early as 1 or 3 days after IRI. The arrows indicate impaired/necrotic tubules. The lower panel shows semi-quantification results of the injured tubules (IRI vs. sham). Ten randomly chosen images in each group were used for the quantification. ***, p < 0.001. **C**. Immunofluorescence detection of PBT-Fc (with DAPI counterstaining) in acutely injured kidneys (1 h after IRI) with a range of severity (20 min, 30 min or 45 min IRI). Sham kidneys from unclamped contralateral kidneys were used as controls, which showed relatively even level of PBT-Fc staining at tubular basement membrane in a mesh-like pattern (middle panels). In contrast, the IRI kidneys all had areas of loss-of-signal (between dotted lines and arrows), suggesting nephron dysfunctions either in glomerular filtration or in tubular reabsorption, or in both. IRI severity seemed correlate with the degree and extend of loss-of-tracer incorporation.

We then set out to image additional experimental models of mice. First, with the same unilateral IRI model (with 30 min clamping) we scanned the earlier time points at 1 day and 3 days of acute phase of injury (Figure 5A). Radiotracer leakage into the bladder was more prominent than after 21 days of recovery (compare Figure 5A with Figure 4B). Moreover, there was still partial signal retention in the injured kidney (Figure 5A), suggesting filtration capacity and tubular activity remained early on during ischemia recovery and then possibly progressed to functional loss as seen in Figure 4. Histological analysis indicated tubular injury (~ 40%) without interstitial fibrosis during this acute injury stage (Figure 5B). It is conceivable that these injured kidneys were still partially functional in terms of filtering tracer from blood and being able to retain the tracer in their still-intact proximal tubules.

We were interested in understanding the resolution of [^99m^Tc]-PBT-Fc in revealing nephron impairment. We performed IF staining of the kidney sections using antibody to detect the intrarenal locations of the tracer (Figure 5C). In agreement with the SPECT results of the probe, alternating patterns of IF signal loss in injured kidneys were evident 1 h after the IRI operation to the kidneys (Figure 5C, IRI compared to sham). IF signal loss appeared in a zoned configuration that likely reflected the cluster of nephrons, in contrast to the neighboring zones that still retained tracer signal (borders marked in Figure 5C). Overall, the zones that had lost tracer signals were more prominent in severe IRI kidneys (Figure 5C, compare among 20, 30 and 45 min IRI).

### Nephrotoxicity models imaged with [^99m^Tc]-PBT-Fc by SPECT

**Figure 6:**
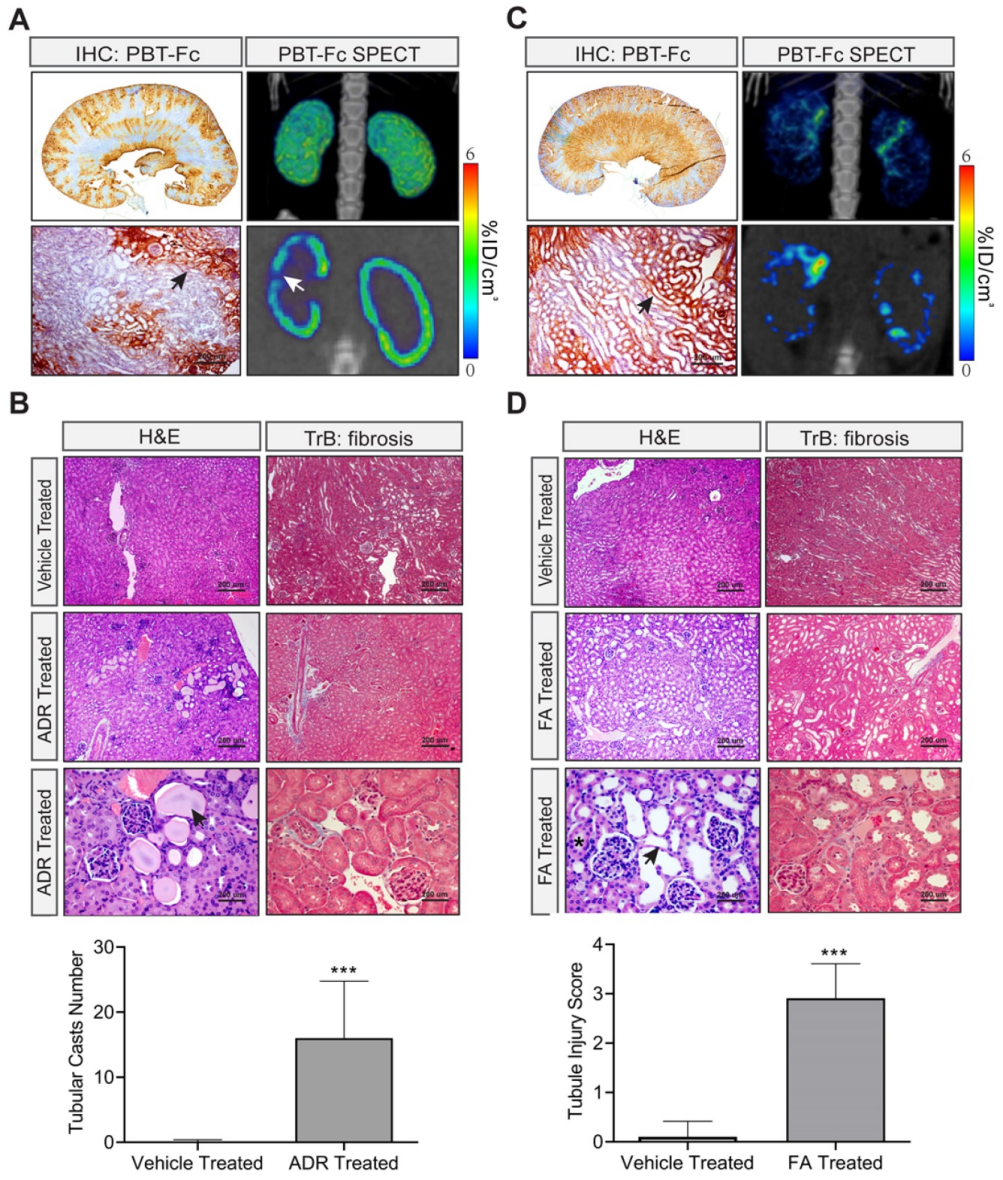
** Models of nephrotoxicity imaged by [^99m^Tc]-PBT-Fc-direct SPECT. A.** Systemic treatment with nephrotoxic drug Adriamycin in an AAN model showed relatively subtle alterations in PBT-Fc-directed SPECT (3D reconstructed view in top left panel). Areas of tracer signal loss was visible in the sectional view (arrow in bottom left panel). IHC detection of the probe (stained in brown) showed a zoned pattern of positive signal along the cortex to medulla directions (top middle showed whole kidney section and bottom middle showed a close-up area with an arrow pointing to preserved kidney functions with staining of the probe). Heatmap scale in %ID/cm^3^. **B.** H&E and Masson trichrome (TrB: fibrosis) staining of the kidneys following Adriamycin (ADR) treatment of the mice. Kidney lesions including tubular protein casts (arrow) were apparent, consistent with the nephrotic characteristics of the AAN model. Bottom panel: Tubular casts were semi-quantified from 10 randomly chosen images in each group (ADR vs. control). **C**. In contrast, the folic acid (FA) nephropathy model of renal tubular injury displayed a drastically altered SPECT pattern of the kidney (compared to normal kidney in Figure 3A). Both 3-D composite and sectional views (left top and left bottom respectively) showed probe signal distributed in a discontinuous pattern. IHC staining also reveal areas of loss-of-signal in the cortex (arrow points tubules with the incorporation of the probe). **D.** H&E and Masson trichrome (TrB: fibrosis) staining showed characteristic lesions from FA-induced tubular obstruction, including intra-tubular crystal formation (asterisk) and tubular dilation and thinning of the tubular epithelial layer (arrow). Tubular injury scoring of the lesions was quantified from 10 randomly chosen images: 0, none; 1, < 25%; 2, 25% - 50%; 3, 50% - 75%; and 4, > 75%. ***, p < 0.001 between FA- and vehicle-treated groups.

Next we tested other types of renal injury from distinct causes, such as Adriamycin-associated nephropathy (AAN) with underlying glomerular (podocyte) injury and proteinuria 29, and folic acid (FA) nephropathy caused by tubular crystal formation and manifested with tissue necrosis 30. As before, in order to better understand the capability of PBT-Fc-directed SPECT in revealing specific renal lesions, we performed non-invasive live imaging study in these two models. We also performed IHC staining to confirm the distribution of the probe and hematoxylin and eosin (H&E) staining to assess the severity of the lesions (Figure 6).

There were relatively subtle observable alterations of acute AAN kidneys revealed by the radiotracer using SPECT despite of the apparent zoned distribution of the tracer detected by IHC (Figure 6A). H&E staining and Masson trichrome staining confirmed the presence of tubular protein casts and relatively normal glomerulus morphology that were consistent with the nephrotic nature of the model (Figure 6B).

In stark contrast, the FA nephropathy kidneys displayed striking SPECT abnormalities as visualized by [^99m^Tc]-PBT-Fc tracer (Figure 6C). The kidneys lost continuity of its cortical tracer retention as the radio signals were distributed in a scattered pattern. IHC also showed zonal loss of tracer signals, while H&E staining and Masson trichrome staining confirmed tubular injuries in the model with dilated tubular lumens, indicating obstruction (Figure 6D). This was consistent with the expectation that FA caused patterned signal loss, due to possible glomerular filtration or tubular reabsorption defects, or both.

### Chronic hypertension kidney disease imaged by [^99m^Tc]-PBT-Fc and SPECT scanning

**Figure 7:**
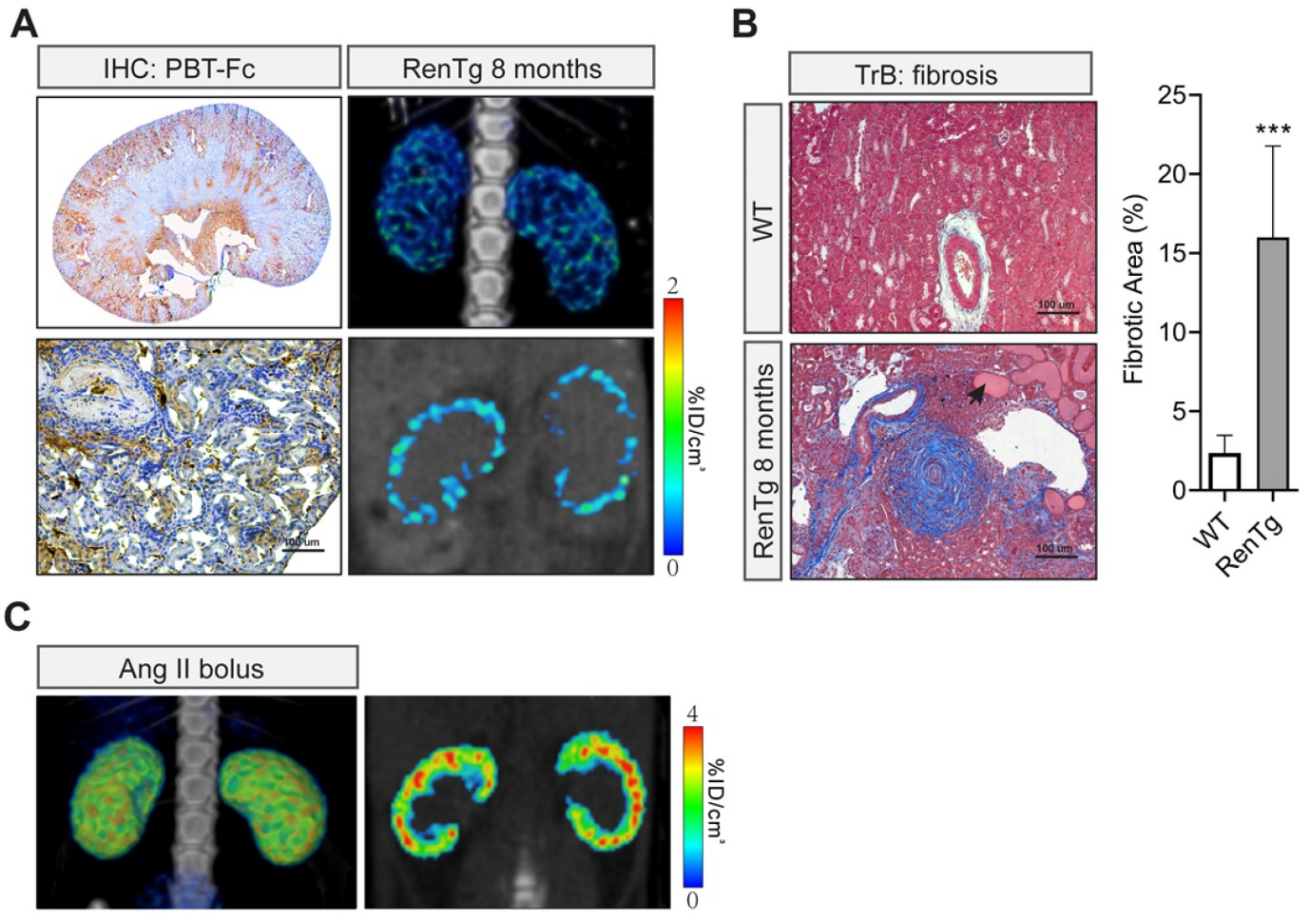
** Non-invasive [^99m^Tc]-PBT-Fc-direct SPECT revealed renal lesions from chronic hypertension. A.** In a chronic hypertensive disease model of RenTg/MK with RAAS over-activation, the eight months old mouse displayed the loss of the tracer signal in regions of the kidney as shown in SPECT (left panels. Top: 3D composite; bottom: section view). Distribution of tracer signal was highly discontinuous. IHC detected fragmented distribution of PBT-Fc (in brown) in whole-kidney (top right) and close-up (bottom right) views. Heatmap scale in %ID/cm^3^. **B.** Masson trichrome staining (TrB: fibrosis) of the kidney sections showed lesions consistent with those of chronic hypertensive injury to the kidney, including onion peels-like fibrosis surround the renal arteries, tubular protein casts (arrow), and extensive inflammatory infiltration. Tissue fibrosis was semi-quantified from 10 randomly chosen images in each group. ***, p < 0.001. **C**. Acute intravenous injection of a mouse with a bolus dose of angiotensin II (Ang II) did not incur the same renal damage as in the chronic hypertensive model in A.

In addition to those artificial models, we imaged chronic hypertensive kidney disease using non-invasive SPECT in conjunction with our renal-targeted [^99m^Tc]-PBT-Fc tracer. Previously, we and others studied a genetic model of renin-angiotensin-system (RAS) over-activation 21. The transgenic mouse, referred to as RenTg/MK, over-expresses a *Renin* transgene and has elevated blood pressure since born. Adult mice develop proteinuria with underlying lesions that are consistent with hypertensive kidney disease. In both 3-dimentional and sectional views (Figure 7A**,** top and bottom right), discontinued presence of radiotracer signal across renal cortex was obvious. IHC also showed zoned staining pattern along the cortex-medulla axis, consistent with the clustered pattern of SPECT signal of the radiotracer. Under higher magnification, tracer staining signals appeared in some areas of the cortex but were absence in regions of fibrosis and/or inflammatory infiltration (Figure 7A, bottom left). Also, as expected, Masson trichrome staining revealed expected lesions of hypertensive kidney disease, including onion peel-like fibrosis and tubular protein casts as seen in CKD (Figure 7B). To ascertain the loss of tracer signals in some areas of the kidney was not due to nephron's response to high angiotensin II in the model, we performed control study of kidneys following short term exposure to a bolus of angiotensin II. Live SPECT with [^99m^Tc]-PBT-Fc tracer did not show alterations of cortical activities (Figure 7C).

### Enabling separate measurements of GFR of individual kidneys

**Figure 8:**
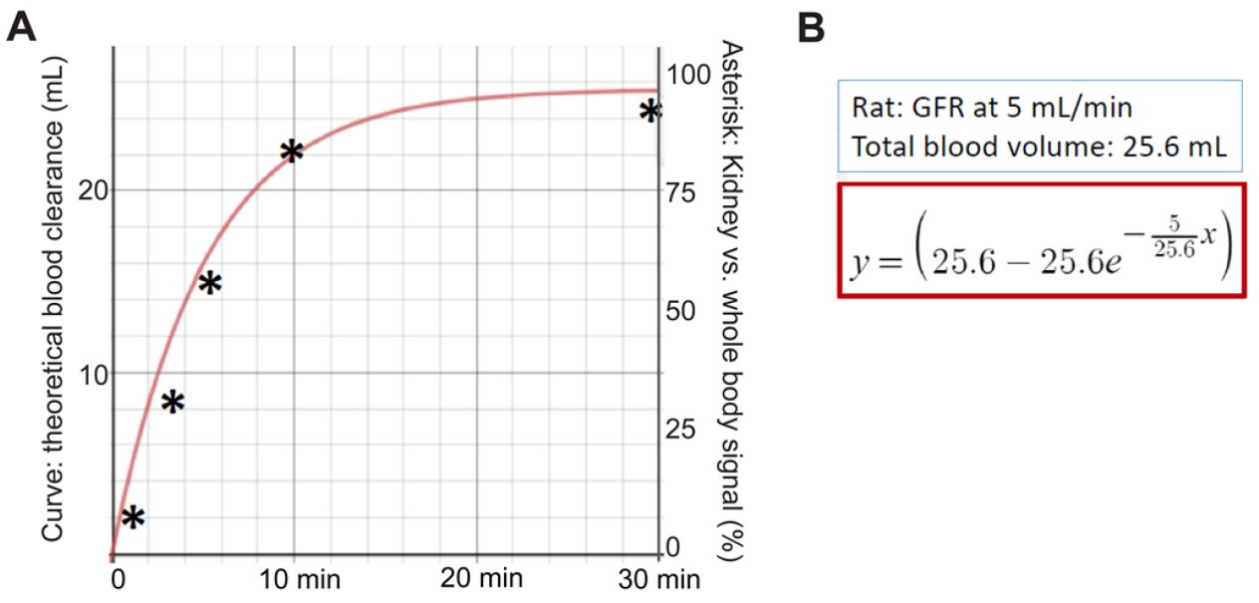
** Glomerular filtration rate (GFR) measured by [^99m^Tc]-PBT-Fc whole-body scintigraphy of rat**. **A**. Based on Figure 1D, the accumulation of PBT-Fc radio signal in the kidney as percentage of the whole-body level was calculated at 1, 3, 5, 10- and 30- min time points (Asterisks: *y*-axis values). The general trend followed the theoretical tracer clearance curve based on an assumption that tracers were completely sequestered in the kidney after filtered at the glomerulus. **B**. The theoretical curve was plotted based on the assumption of GFR = 5 mL/min and the rat had a total blood volume of 25.6 mL (see Methods). The equation for calculating the *y*-axel values was based on these parameters.

We explored additional applications of the new tracer for calculating glomerular filtration rate (GFR). It should be noted that when a passive tracer such as DTPA is used the GFR is calculated from the clearance rate of the chemical from blood into urine 31-33. Therefore, it is practically impossible to separately attribute GFR to individual kidneys including renal grafts in case of uneven functional capacities exist between two kidneys. In theory, the new PBT-Fc probe will enable the calculation of GFR of individual kidneys via an alternative strategy. Instead of calculating blood clearance rate, by following the kinetics of [^99m^Tc]-PBT-Fc being captured in individual kidneys, we derived GFR based on the rate of signal accumulation in the kidneys. This was under the assumption that renal sequestration of the probe was the collective actions of glomerular filtration and tubular reabsorption (Figure 1C). When we based on the dynamic renal accumulation of tracer signal in Figure 1D to calculate GFR, the sample point trend matched the theoretical filtration dynamics (Figure 8) (in Methods).

## Discussion

Conventional renal nuclear imaging uses passive chemical tracers that lack specificity and produce poor image quality. We followed a targeted design to construct a recombinant protein tracer referred to as PBT-Fc. The new tracer is a chimeric fusion comprised of segments of VEGFA and IgG1 respectively. Through a sequence of intrarenal movements mediated by cognate FcRn receptor and then by tubular basement membrane, the tracer is sequestered in the kidney with virtually no surrounding tissue background or urinary loss. This total renal fixation of the tracer permitted extended radionuclide signal acquisition time, from ~ 15 s in conventional radioisotope renography of constantly moving tracer signal, to 15 - 20 min using [^99m^Tc]-labeled PBT-Fc that was fixed at the renal cortex. Again, we emphasized on the critical importance of target sequestration for aiding signal acquisition of SPECT for high-resolution imaging. To our limited knowledge, our new renal-targeting probe is the first-in-class radiopharmaceutical to achieve super long-lasting and kidney-specific sequestration, perhaps in par with the performance of Iodine (i.e. ^123^I and ^131^I) in the thyroid scan and phosphorus-32 (i.e. [^32^P]-MDP) in bone scintigraphy. Additional mouse studies showed PBT-Fc remained detectable, albeit at low intensity, at renal tubular basement membrane 3 days after injection and was completely cleared after 7 days (Figure S6). Benefited from long renal sequestration of the radiotracer with little urinary loss due to protein degradation, the new tracer, when used in conjunction with SPECT, revealed the kidney lesions at unprecedented submillimeter resolution.

As noted above, conventional renal tracers such as DMSA and GHA also have some renal accumulations and therefore are used in conjunction with SPECT to image functional renal mass 34, 35. Our results showed a very modest retention of DMSA in organs including the lung and the kidney (longer organ retention than MAG3: Figure S5). However, the precise distribution of these agents at the cellular level in the kidney remains uncertain 36, and their clinical indications have mostly been developed empirically through testing. By contrast, the new PBT-Fc tracer was designed to participate in intended renal physiologic processes such as glomerulus filtration and tubular reabsorption (i.e. via FcRn-mediated transcytosis). In mice, through immunohistochemistry and immunofluorescence detections of the tracer, the precise intrarenal route and ultimate location of fixation of the tracer were determined. Therefore, [^99m^Tc]-PBT-Fc-directed renogram imaging illustrates the spatial pattern of accumulated filtration activity.

From a practical perspective, most serum biomarkers for measuring renal function, such as serum urea and creatinine, significantly trail the decline of kidney function. Moreover, these serum biomarkers do not always correlate with the degree of irreversible kidney damage such as fibrosis. Therefore, new methods that can detect anatomical lesions of the kidney in real time by non-invasive nuclear imaging are highly desirable 37. In PBT-Fc, we emphasize that the Fc module of the tracer directs protein reuptake at the proximal tubule through a different mechanism than protein sorting by megalin and cubilin. Megalin and cubilin direct their respective ligands such as β2M to lysosomal degradation in tubular cells 38, leading to the release and subsequent urinary excretion of any radionuclide attachments. In contrast, FcRn mediates transcytotic recycling that can circumvent lysosomal degradation of its 'cargo' proteins such as albumin and IgG 39. Injected at doses as high as 250 μg/kg.BW to mice and rats, the tracer seemed to be well tolerated without noticeable systemic or renal abnormalities (not shown). However, it is important to point out that neutralizing antibodies are expected to develop that will affect repeated doses to the same animal. This is primarily because we used human protein sequences from VEGFA and IgG1 to construct PBT-Fc. It remains to be determined whether the tracer will still elicit antibody response in human, which would then require further modification of the junctional sequence between PBT and Fc to reduce its antigenicity.

Furthermore, as compared to the standard of care chemical tracers for renal scan, recombinant tracers have their disadvantages. These include the higher costs associated with production and storage of biologics, expected short shelf-life of protein drugs, complex labeling procedures with expected low levels of ^99m^Tc incorporation, as well as unproven safety status that needs to be assessed in clinical trials.

In summary, we designed and tested a novel protein-based radiotracer for high-performance SPECT imaging of the kidney. With its exclusively targeted sequestration in the renal parenchyma, while having virtually no signal loss from urinary excretion, the successful preclinical testing of this PBT-Fc tracer represents a conceptual advancement in radiopharmaceutical design for renal imaging.

## Supplementary Material

Supplementary figures.

## Abbreviations

SPECT: single-photon emission computerized tomography
IPTG: isopropyl β-d-1-thiogalactopyranoside
PFA: paraformaldehyde
FcRn: neonatal Fc receptor
