# Supplementary material for: Targeted design of a recombinant tracer for SPECT renal imaging
Source: Theranostics. 2021 Aug 27;11(18):9118–32. doi: 10.7150/thno.60132 (PMC8419037; doi:10.7150/thno.60132)
Supplement: Supplementary file 1 — Supplementary figures. [file thnov11p9118s1.pdf]

Supplementary Figures

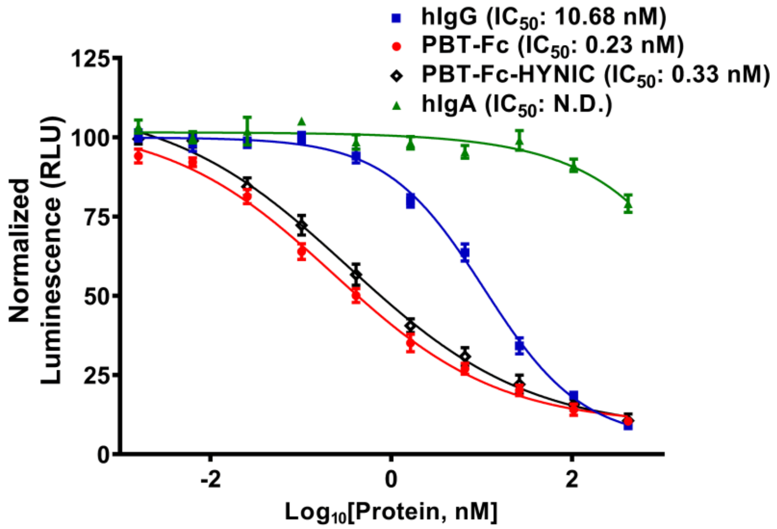

Figure S1. PBT-Fc and PBT-Fc-HYNIC specifically bind FcRn. IC_{50} values were calculated based on the concentrations needed to inhibit 50% reporter activity (see Methods). Data are shown as the mean ± SD from triplicate experiments. The PBT-Fc and PBT-Fc-HYNIC showed comparable affinity to FcRn. The human IgG and IgA were used as positive and negative controls, respectively.

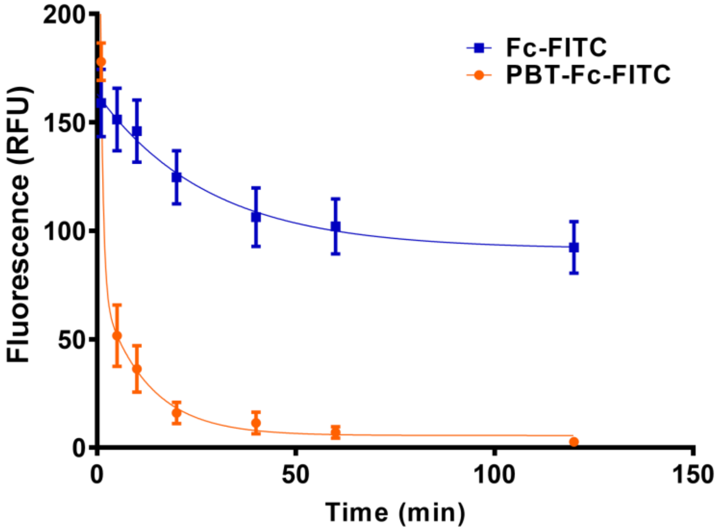

Figure S2. Pharmacokinetics of FITC-labeled Fc- and PBT-Fc in blood circulation. Serum samples were collected from mice (n = 3 for each group) at different timepoints after receiving *i.v.* injection of FITC-labeled Fc or PBT-Fc. Fluorescence intensity was measured at Ex485/Em530 nm (for detecting FITC). Fc-FITC showed relatively steady blood retention after a ~ 40 min tissue redistribution phase, whereas PBT-Fc-FITC showed faster clearance (~ 20 min) from circulation.

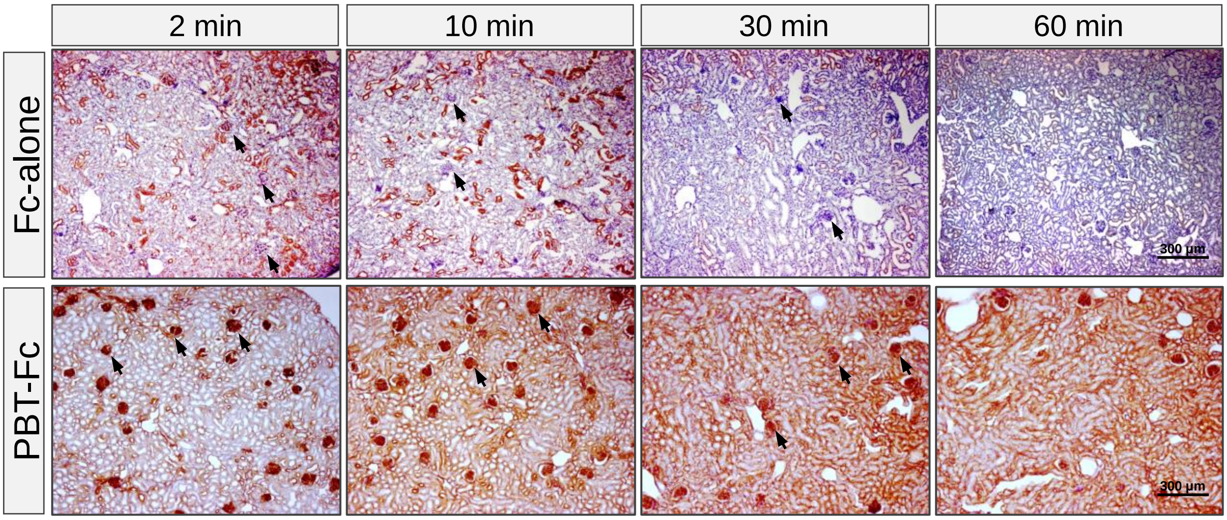

Figure S3. Contrasting differences in dynamic intrarenal redistribution of Fc-alone and PBT-Fc during the first hour after injection. Kidney sections of mice were collected from 2 min to 60 min after injection of the probes. The sections were stained using anti-Fc antibody to detect the probes (brown IHC signals on the background of Hematoxylin counterstaining of cell nuclei). Examples of the glomerulus are pointed by arrows. The Fc-alone probe showed a glomerulus-to-proximal tubule transition in the early time sequence, and then disappeared after 10 min. In contrast, early staining of PBT-Fc was concentrated in the glomerulus, followed by a gradual transition to more prominent tubular signals that sustained beyond 60 min. Although not quantitative, the overall signal intensity of the PBT-Fc in the kidney generally increased from 2 min to 10 min, and thereafter changed very little, consistent with radio-scintigraphy findings in Figure 1D.

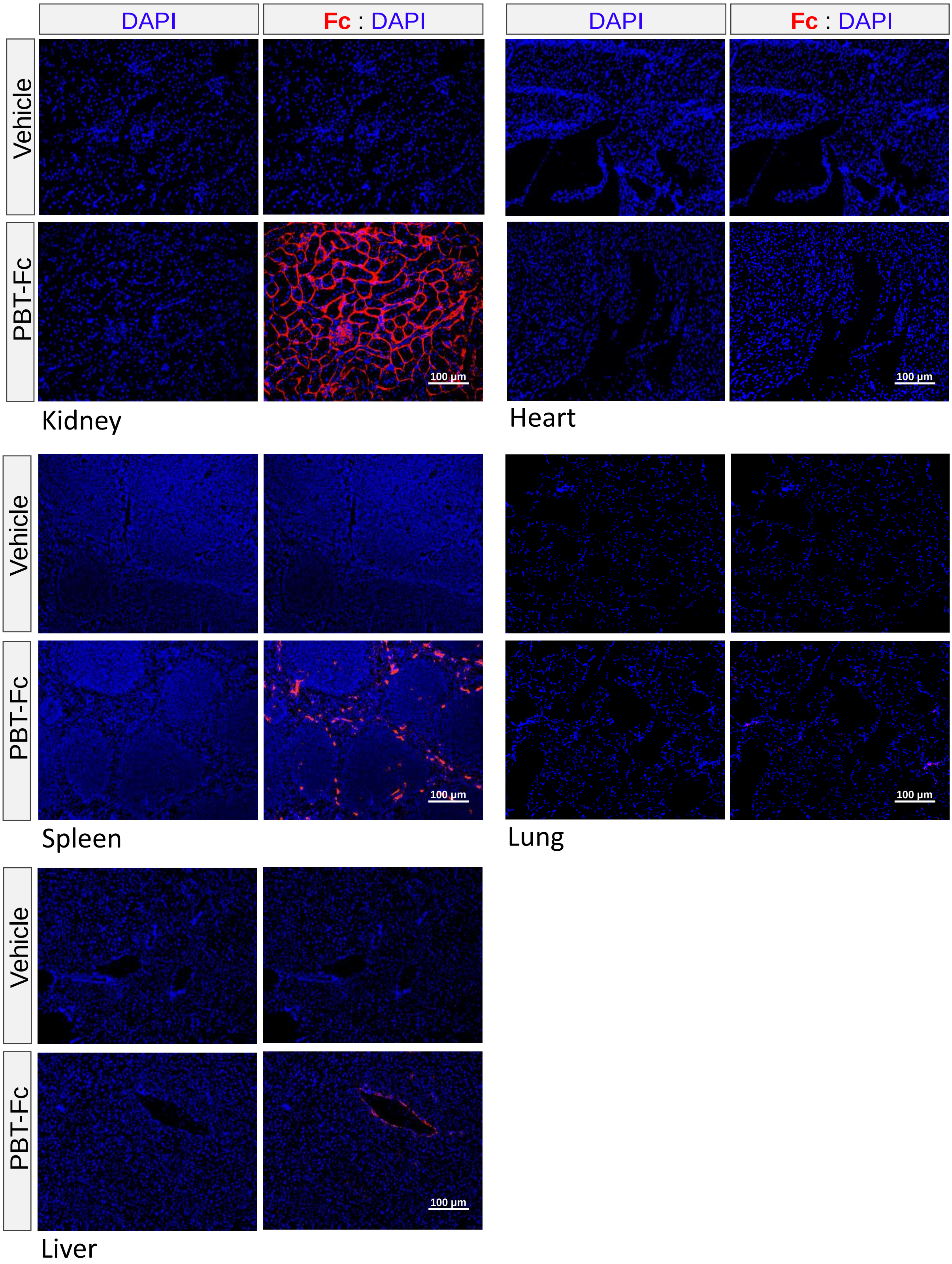

Figure S4. The steady-phase biodistribution of PBT-Fc in organs as detected by immunofluorescence staining. 6 h following *i.v.* injection of either vehicle or PBT-Fc in mice, the kidney, the heart, the spleen, the lung and the liver were harvested for immunofluorescence detection of PBT-Fc (in red: stained with anti-Fc antibody). DAPI staining for the nuclei was used as guiding counterstaining (in blue). No Fc signals were detected in vehicle controls. The kidney showed a strong mesh-like pattern of PBT-Fc that matched the locations of tubular basement membrane (TBM) at the basal side of the DAPI-positive lining of tubular epithelial cells. In the spleen and liver, weaker PBT-Fc signals were detected along the follicle boundaries and vascular wall, respectively. The heart and the lung were completely absent of PBT-Fc sequestration in tissues.

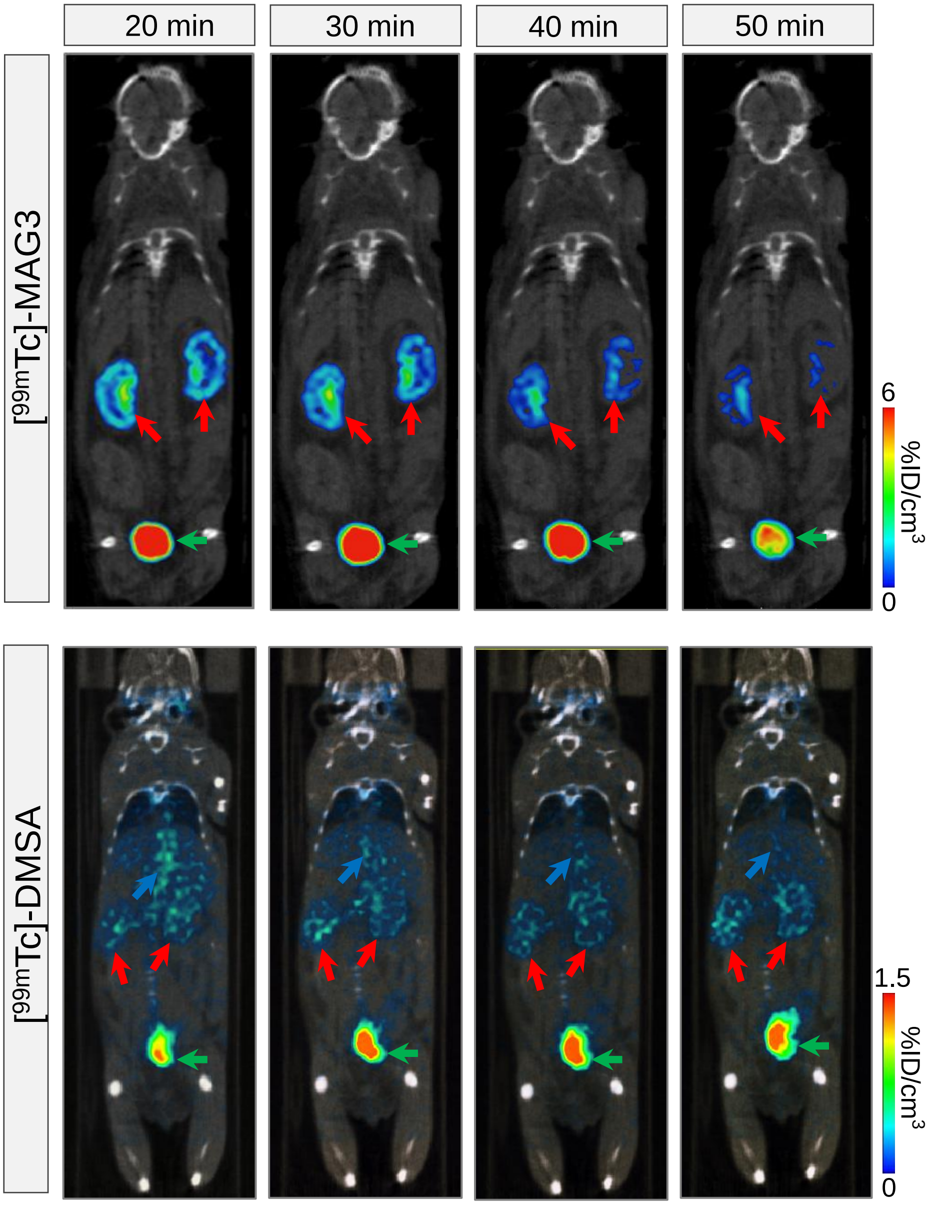

Figure S5. Dynamic SPECT scanning of mice using standard of care [^{99m}Tc]-MAG3 and [^{99m}Tc]-DMSA radiotracers. From 20 min after *i.v.* injection of the radiotracers , the mice were scanned by SPECT with an acquisition time of 10 min. A total of 4 scans were performed to each mouse. Six mice were used for each tracer (n = 6) and representative SPECT images are shown (signal intensity levels are indicated by "heatmaps": low-to-high levels represented by the scale bar). Overall, both tracers followed fast urinary excretion with most signals accumulated in the bladder (green arrows). While both tracers also illuminated the kidney (red arrows), DMSA stayed a longer time in the kidney, remained detectable at 50 min, albeit already much weaker than its intensity in the bladder. In contrast, MAG3 quickly filled the kidney, more concentrated in the renal pelvis. However, little kidney intensity remained after 50 min. The results are consistent with the expectation of [^{99m}Tc]-MAG3 and [^{99m}Tc]-DMSA to show only transient retention in kidney with fast urinary excretion. In addition, consistent with the understanding of DMSA to have better tissue/organ retention than MAG3, DMSA also showed signals in the lung/heart (blue arrows) at the earlier timepoints (most prominent before 30 min).

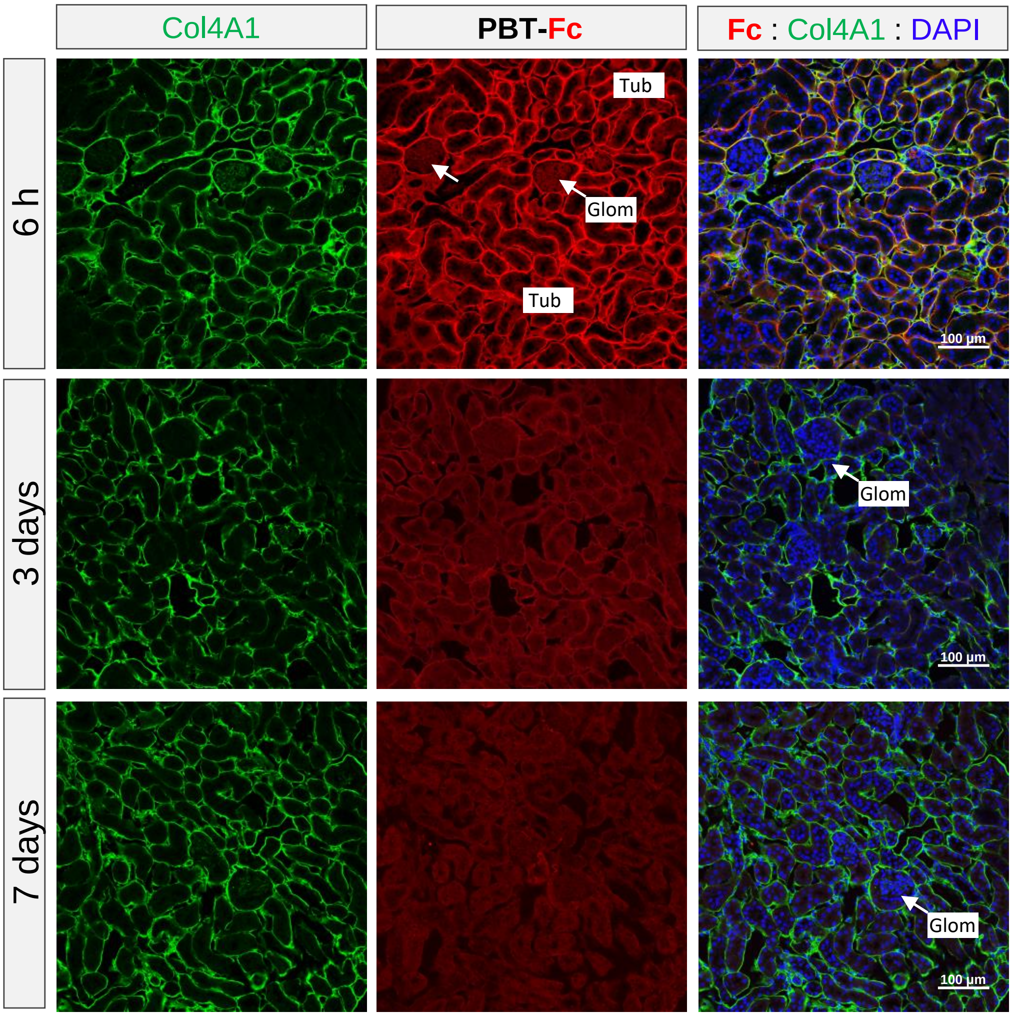

Figure S6. Long-lasting renal sequestration of PBT-Fc at the tubular basement membrane. Mice received a single bolus dose of PBT-Fc was subjected to immunofluorescence detection of the probe (red) in kidney cortex. Col4A1 and DAPI were counterstains. Prominent TBM signal of PBT-Fc staining was evident at the 6 h timepoint. By 3 days, weak PBT-Fc signals in association with the TBM remained detectable. By 6 days after injection of PBT-Fc, no specific TBM-associated staining was detected, indicating complete renal clearance of the probe.
